# Dr. James B. Hunter

**Published:** 1889-08

**Authors:** 


DR. JAMES B. HUNTER, one of the surgeons of the Woman's Hospital of the State of New York, died at his home in New York City, June 10, 1889, aged 52 years. Dr. Hunter was a man of rare accomplishments, a conspicuous light in his chosen specialty, rendered distinguished service in the medical staff of the army during the late war of the rebellion, and was a member of the Military Order of the Loyal Legion at the time of his death.

